# The DNABII family of proteins is comprised of the only nucleoid associated proteins required for nontypeable *Haemophilus influenzae* biofilm structure

**DOI:** 10.1002/mbo3.563

**Published:** 2017-12-12

**Authors:** Aishwarya Devaraj, John Buzzo, Christopher J. Rocco, Lauren O. Bakaletz, Steven D. Goodman

**Affiliations:** ^1^ Center for Microbial Pathogenesis The Research Institute at Nationwide Children's Hospital and The Ohio State University College of Medicine Columbus OH USA

**Keywords:** extracellular matrix, HU, IHF

## Abstract

Biofilms play a central role in the pathobiology of otitis media (OM), bronchitis, sinusitis, conjunctivitis, and pneumonia caused by nontypeable *Haemophilus influenzae* (NTHI). Our previous studies show that extracellular DNA (eDNA) and DNABII proteins are essential components of biofilms formed by NTHI. The DNABII protein family includes integration host factor (IHF) and the histone‐like protein HU and plays a central role in NTHI biofilm structural integrity. We demonstrated that immunological targeting of these proteins during NTHI‐induced experimental OM in a chinchilla model caused rapid clearance of biofilms from the middle ear. Given the essential role of DNABII proteins in maintaining the structure of an NTHI biofilm, we investigated whether any of the other nucleoid associated proteins (NAPs) expressed by NTHI might play a similar role, thereby serving as additional target(s) for intervention. We demonstrated that although several NAPs including H‐NS, CbpA, HfQ and Dps are present within the biofilm extracellular matrix, only the DNABII family of proteins is critical for the structural integrity of the biofilms formed by NTHI. We have also demonstrated that IHF and HU are located at distinct regions within the extracellular matrix of NTHI biofilms formed in vitro, indicative of independent functions of these two proteins.

## 1 INTRODUCTION

*Haemophilus influenzae* is an often pathogenic, fastidious Gram‐negative, facultative anaerobe in the *Pasteurellaceae* family. Some strains of *H. influenzae* are encapsulated and can be classified into six serotypes (a‐f) on the basis of their distinct capsular antigens (Pittman, 1931). Nontypeable *Haemophilus influenzae* (NTHI) strains are nonencapsulated and cannot be serotyped by conventional means. While the introduction of *Haemophilus influenzae* type b (Hib) conjugate vaccine more than 20 years ago resulted in a rapid reduction of invasive infections caused by *H. influenzae type b*, there has been a concomitant 6‐fold increase in incidence of invasive infections caused by NTHI strains (Gessner & Adegbola, 2008; MacNeil et al., 2011; Resman et al., 2011; Tsang et al., 2007).

NTHI strains reside as commensal bacteria in the human nasopharynx and are opportunistic pathogens that cause several noninvasive infections, including otitis media (OM), sinusitis, conjunctivitis, bronchitis, exacerbations of chronic obstructive pulmonary disease (COPD), and pneumonia. NTHI also causes invasive infections, including sepsis and meningitis in neonates (MacNeil et al., 2011). NTHI accounts for more than 40% of incidence of acute OM (Haggard, 2008), 44–68% of cases of conjunctivitis (Buznach, Dagan, & Greenberg, 2005; Chawla, Kellner, & Astle, 2001), 41% of cases of sinusitis (Brook, 2007), 81% of cases of bronchitis (Butt et al., 1990), >90% of exacerbations of COPD (Sethi & Murphy, 2001; Sethi et al., 2008), up to 40% of cases of pneumonia (De Schutter et al., 2011) and is the predominant pathogen of both chronic and recurrent OM (Haggard, 2008).

The ability of NTHI to form biofilms plays a major role in chronic and recurrent infections and has been implicated in the failure of antibiotic therapy, the first‐line of treatment for bacterial infections (Bakaletz, 2012; Erwin & Smith, 2007; Hall‐Stoodley et al., 2006; Leroy et al., 2007; Morris, 2007; Post, Hiller, Nistico, Stoodley, & Ehrlich, 2007; Starner, Zhang, Kim, Apicella, & McCray, 2006). It has been well established that bacteria in a biofilm are 1,000‐fold more resistant to antimicrobial agents compared to the bacteria in planktonic state (Devaraj, Justice, Bakaletz, & Goodman, 2015; Kaji, Watanabe, Apicella, & Watanabe, 2008; Slinger et al., 2006; Starner, Shrout, Parsek, Appelbaum, & Kim, 2008). The chronic and recurrent nature of biofilm‐mediated diseases demands extensive use of both narrow‐ and broad‐spectrum antibiotics that in turn has led to the evolution of antibiotic‐resistant bacteria (Stool et al., 1994; Williams, Chalmers, Stange, Chalmers, & Bowlin, 1993). Therefore, there is a pressing need for development of novel therapies to impede bacterial pathogenesis mediated by biofilms.

We have previously shown that NTHI forms a robust biofilm in vitro and in vivo in the chinchilla middle ear during experimental OM (Goodman et al., 2011; Idicula et al., 2016; Jurcisek & Bakaletz, 2007). NTHI, amongst most other human pathogens, incorporate an abundant amount of extracellular DNA (eDNA) that is arranged in an intricate lattice‐like network within the extracellular matrix (ECM) (Devaraj et al., 2015; Gustave, Jurcisek, McCoy, Goodman, & Bakaletz, 2013; Idicula et al., 2016; Jurcisek & Bakaletz, 2007; Novotny, Amer, Brockson, Goodman, & Bakaletz, 2013). The organization of eDNA within the ECM of bacterial biofilms bears a striking resemblance to the structure of preferred DNA substrates of the DNABII proteins (Kamashev & Rouviere‐Yaniv, 2000). Integration host factor (IHF) and histone‐like protein (HU) are the two known nucleoid associated proteins (NAPs) in the DNABII protein family, which bind to DNA with high affinity, bend DNA, and play a critical role in intracellular bacterial nucleoid structure and function (Browning, Grainger, & Busby, 2010). We have demonstrated in three distinct animal models that IHF and HU are part of the ECM of the biofilms formed by pathogens of the oral cavity and respiratory tract and play a key role in maintaining the lattice‐like structure of the eDNA by binding to the vertices of each crossed strand of eDNA (Freire et al., 2016; Goodman et al., 2011; Gustave et al., 2013; Idicula et al., 2016; Novotny, Jurcisek, Goodman, & Bakaletz, 2016). We have also demonstrated that removal of DNABII proteins with DNABII protein specific antibodies disrupt in vitro preformed biofilms of several human pathogens (Brandstetter, Jurcisek, Goodman, Bakaletz, & Das, 2013; Brockson et al., 2014; Goodman et al., 2011; Novotny et al., 2013, 2016; Rocco, Davey, Bakaletz, & Goodman, 2016), induce total collapse of the biofilm structure and promotes subsequent clearance of resident bacteria in vivo during NTHI‐induced experimental OM, *Pseudomonas aeruginosa*‐induced lung infection, and resolve inflammation during *Aggregatibacter actinomycetemcomitans‐*induced experimental peri‐implantitis (Freire et al., 2016; Novotny et al., 2016). DNABII‐specific antibodies are also highly effective in dispersal of polymicrobial biofilms contained within sputum solids recovered from pediatric cystic fibrosis patients (Gustave et al., 2013). Collectively, our previous studies have demonstrated that DNABII proteins serve as viable targets for the development of a highly effective, broad‐spectrum therapeutic to resolve bacterial diseases mediated by biofilms.

While our studies to date show that DNABII proteins are critical for the structural integrity of the ECM of bacterial biofilms, we have yet to determine if other NAPs also play a role in maintaining the structure of the ECM and therefore represent additional targets for development of therapeutics for removal of bacterial biofilms. The NTHI genome, including that of strain 86‐028NP which we use as model organism herein, has genes that encode for several NAPs including three alleles that encode the aforementioned DNABII proteins as well as Dps (DNA binding protein from starved cells), H‐NS (histone‐like nucleoid structuring protein), HfQ (host factor for phage Q_β_), Fis (factor for inversion stimulation), CbpA (curved DNA binding protein A) and CbpB (curved DNA binding protein B) (Harrison et al., 2005). The DNABII family is further subdivided into HU and IHF with the former being ubiquitously expressed in all eubacteria and the latter expressed only in proteobacter Gram‐negative species. The binding specificities and affinity of *E. coli* NAPs to DNA are well characterized; IHF, Fis, and CbpB bind to a specific DNA sequence while the remainder of the NAPs bind DNA in a sequence independent manner (Azam & Ishihama, 1999). In addition, CbpA, H‐NS and HfQ preferentially bind curved DNA, and DNABII proteins preferentially bind to pre‐bent DNA structures (Azam & Ishihama, 1999; Kamashev & Rouviere‐Yaniv, 2000). Throughout this work, we will refer to IHF and HU as DNABII proteins whereas all other NAPs will be referred to as non‐DNABII proteins.

In this study, while we found multiple NAPs including IHF, HU, H‐NS, Dps, HfQ and CbpA within the ECM of biofilms formed in vitro by NTHI, only IHF and HU were required to maintain the structure of the biofilm; NAPs of the non‐DNABII protein family could be depleted from an NTHI biofilm with no measurable effect on the biofilm structure. These data suggested that DNABII proteins were the dominant structural NAPs and reinforced the concept that they are solely responsible for maintaining the structural integrity of biofilms formed by NTHI.

## 2 EXPERIMENTAL PROCEDURES

### 2.1 Bacterial strains

NTHI strain 86‐028NP isolated at Nationwide Children's Hospital from the nasopharynx of a child with chronic otitis media was used in this study. NTHI strain 86‐028NP has been sequenced (Harrison et al., 2005) and extensively characterized (Bakaletz, Leake, Billy, & Kaumaya, 1997; Bakaletz et al., 1988; Holmes & Bakaletz, 1997; Mason, Munson, & Bakaletz, 2003). NTHI strain 86‐028NP deficient in Dps (NTHI *Δdps*) was described in (Harrison, Baker, & Munson, 2015).

### 2.2 Purification of NTHI NAPs and generation of polyclonal antisera directed against each of the NAPs

Tagless recombinant NAPs were generated, using the IMPACT kit (New England Biolabs) as previously described (Novotny et al., 2016). Dps was PCR amplified from NTHI strain 86‐028NP genomic DNA using the following oligonucleotides: 5′‐ GGTGGTCATATGTCAAAAACATCAATCGGAC – 3′ and 5′ – GGTGGTTGCTCTTCCGCAATTATGGCAAGTTTG – 3′. HfQ was PCR amplified from NTHI strain 86‐028NP genomic DNA using the following oligonucleotides 5′ ‐ GGTGGTCATATGGCAAAAGGACAATCTTTAC – 3′ and 5′ – GGTGGTTGCTCTTCCGCATGCCTCTGCTTGAGTTTC – 3′. H‐NS was PCR amplified from NTHI strain 86‐028NP genomic DNA using the following oligonucleotides 5′ ‐ GGTGGTCATATGAACGAATTAGTAAGAGGTTTA – 3′ and 5′ – GGTGGTTGCTCTTCCGCATGCAATTTCGAAATCAGA – 3′. CbpA was PCR amplified from NTHI strain 86‐028NP genomic DNA, using the following oligonucleotides 5′ – GGTGGTCATATGGCAAAAAAAGATTAC – 3′ and 5′ – GGTGGTTGCTCTTCCGCATTTGTCAGATTTACCCAA – 3′. The PCR products were cloned into pTXB1 vector as described in (Novotny et al., 2016). The constructs were transformed into the *E. coli* expression strain ER2566 (New England Biolabs) to generate strains SG911 (HfQ), SG912 (CbpA), SG913 (Dps), SG914 (H‐NS), SG1096 (IHFA), SG1097 (IHFB), and SG1093 (HU). LB agar containing 100 μg ampicillin/ml was used for selection. The NAPs were overexpressed and purified on a chitin resin column as described (Novotny et al., 2016). IHFA, IHFB, HU, HfQ, and CbpA were further purified on a HiTrap^®^ Heparin HP column (GE Healthcare) equilibrated in 10 mmol/L sodium phosphate pH 7, 200 mmol/L NaCl. The bound protein was eluted with 25 column volumes of linear gradient of elution buffer (10 mmol/L sodium phosphate buffer pH 7, 2M NaCl). The protein was concentrated in a centrifugal filter (3000 MWCO) and dialyzed in storage buffer (50 mmol/L Tris pH 7.4, 600 mmol/L KCl, 1 mmol/L EDTA, 10% glycerol) and stored at −80°C. H‐NS was also further purified with a HiTrap^®^ Heparin HP column equilibrated in 10 mmol/L sodium phosphate pH 7, 500 mmol/L NaCl. Dps was further purified with ammonium sulphate (30% saturation) and clarified at 25,000 g for 15 min at 4°C. The protein was concentrated in a centrifugal filter (3,000 MWCO) and dialyzed in a storage buffer (50 mmol/L Tris pH 7.4, 600 mmol/L KCl, 1 mmol/L EDTA, 10% glycerol) and stored at −80°C. Polyclonal antiserum against each of the purified recombinant NAPs was generated at Spring Valley Laboratories (Sykesville, MD).

### 2.3 Purification of IgG from serum

We purified IgG from rabbit polyclonal antiserum directed against NAPs (anti‐IHF, anti‐HU, anti‐H‐NS, anti‐HfQ, anti‐CbpA and anti‐Dps) with HiTrap Protein G HP column (GE Healthcare) according to the manufacturer's instructions. The IgG‐enriched fraction was dialyzed in 150 mmol/L Tris pH 7.4, 150 mmol/L KCl. IgG concentration was estimated by Pierce^™^ BCA protein assay kit (Fisher Scientific) and confirmed by SDS‐PAGE gel. IgG was stored at −80°C in 150 mmol/L Tris pH 7.4, 150 mmol/L KCl, 10% glycerol.

### 2.4 In vitro biofilm assay

For in vitro biofilm analysis, NTHI strain 86‐028NP was cultured on chocolate agar for 18–20 hr at 37°C, in a humidified atmosphere containing 5% CO_2_, and then suspended in a Brain Heart Infusion broth (BHI) supplemented with 2 μg heme/ml and 2 μg β‐NAD/mL (sBHI) to an OD of 0.65 at 490 nm. Cultures were diluted (1:6) in sBHI broth and then incubated statically at 37°C, 5% CO_2_ until reaching an OD_490 nm_ of 0.65. The cultures were diluted 1:2,500 in sBHI, and 200 μl of this suspension was inoculated into each well of an 8‐well chamber glass slide (Fisher Scientific). After 16 hr of incubation at 37°C, 5% CO_2_, the medium was replaced with fresh medium. After an additional 8 hr incubation period, the medium was replaced again as described above and the chamber slides were incubated for an additional 16 hr as before. At 40 hr, biofilms were either prepared for immunofluorescence (see below) or washed twice with saline (0.9% NaCl) and stained with LIVE/DEAD^®^ stain (Molecular probes, Eugene, OR) according to the manufacturer's instructions. Biofilms were then washed twice in saline and fixed with 1.6% paraformaldehyde, 0.025% glutaraldehyde and 4% acetic acid in 0.1 mol/L phosphate buffer at pH 7.4.

### 2.5 Detection of NAPs within an NTHI biofilm by immunofluorescence

Biofilm formed by NTHI strain 86‐028NP was established on a 8‐well chambered coverglass (Lab‐Tek) for 40 hr as described above in section ‘in vitro biofilm formation’. Unfixed 40 hr biofilms were incubated with 1.5 μg purified polyclonal IgG from rabbits directed against the respective NAPs (anti‐IHF, anti‐HU, anti‐Dps, anti‐H‐NS, anti‐HfQ and anti‐CbpA) in phosphate buffered saline (PBS) for 1 hr at room temperature. The biofilms were washed twice with PBS and then incubated with goat anti‐rabbit IgG conjugated to AlexaFluor^®^ 594 (Molecular Probes) in phosphate buffered saline (PBS) for 1 hr at room temperature. The biofilms were washed twice in PBS and then incubated with DAPI stain (200 μg/ml) in PBS for 10 min. The biofilms were washed once in PBS and then imaged using a ×63 objective on a Zeiss 510 Meta‐laser scanning confocal microscope (Carl Zeiss, Thornwood, NY). Three‐dimensional images were reconstructed with AxioVision Rel. 4.8 (Carl Zeiss). Zeiss image acquisition software was employed to determine the relative fluorescence of anti‐NAP antibody labeling to DAPI. The relative abundance of NAPs was determined by the ratio of the NAP (anti‐NAP labeling) to the fluorescence intensity of DAPI.

### 2.6 Localization of IHF and HU within an NTHI biofilm by immunofluorescence

Unfixed 40 hr NTHI strain 86‐028NP biofilms were labeled with 3.0 μg each of mouse monoclonal antibodies directed against IHFA (anti‐IHFA3) and IHFB (anti‐IHFB2) (Novotny et al., 2016) and guinea pig polyclonal antiserum directed against HU peptide (KKQAKAALEATLDAITASLKEG; anti‐HU_2_) in phosphate buffered saline (PBS) for 1 hr at room temperature. The biofilms were washed twice with PBS and then incubated with goat anti‐mouse IgG conjugated to AlexaFluor^®^ 405 and goat anti‐guinea pig IgG conjugated to AlexaFluor^®^ 488 (Molecular Probes) in phosphate buffered saline (PBS) for 1 hr at room temperature. Bacterial cells were labeled with FilmTracer™ FM^®^ 4–64 (Molecular Probes) according to manufacturer's instructions. The biofilms were imaged, using a x63 objective on a Zeiss 510 Meta‐laser scanning confocal microscope (Carl Zeiss, Thornwood, NY). Three‐dimensional images were reconstructed with AxioVision Rel. 4.8 (Carl Zeiss).

### 2.7 Biofilm disruption assay

Biofilms formed by NTHI strain 86‐028NP were established on a 8‐well chambered coverglass (Lab‐Tek) for 24 hr as described above in section ‘in vitro biofilm formation’. At 24 hr, 30 μg of rabbit preimmune IgG or polyclonal IgG directed against the respective NAPs (anti‐IHF, anti‐HU, anti‐Dps, anti‐H‐NS, anti‐HfQ and anti‐CbpA) was added and incubated for an additional 16 hr. After the final incubation (40 hr total), the medium was removed and the biofilms were washed twice with saline (0.9% NaCl) and stained with LIVE/DEAD^®^ stain (Molecular probes, Eugene, OR) according to manufacturer's instructions. Biofilms were then washed twice in saline and fixed with 1.6% paraformaldehyde, 0.025% glutaraldehyde and 4% acetic acid in 0.1 mol/L phosphate buffer at pH 7.4. The biofilms were imaged, using a x63 objective on a Zeiss 510 Meta‐laser scanning confocal microscope (Carl Zeiss, Thornwood, NY). Three‐dimensional images were reconstructed with AxioVision Rel. 4.8 (Carl Zeiss) and average thickness and biomass were determined by COMSTAT analysis (Heydorn et al., 2000). All in vitro biofilm assays were repeated a minimum of three times on separate days and all individual biofilm assays were done in duplicates on each assay day. Data are presented as mean values ± SEM.

### 2.8 Depletion and exogenous addition of NAPs to biofilms formed by NTHI strain 86‐028NP

NTHI strain 86‐028NP biofilm was established on a 8‐well chambered coverglass (Lab‐Tek) for 40 hr as described above in section ‘in vitro biofilm assay’. For depletion of NAPs, a 1:200 dilution of the polyclonal antiserum directed against the respective DNA binding protein was added at the time of seeding. For exogenous addition of NAPs, 100 nmol/L of the appropriate recombinant protein was added at the time of seeding. CbpA (100 nmol/L) was added at 16 hr. Also, the respective antiserum (1:200) or appropriate recombinant protein (100 nmol/L) was added every time the medium was replaced. Preimmune serum was used as a ‘no depletion control’ for depletion experiments. At 40 hr, the biofilms were washed twice with PBS and incubated with polyclonal antiserum against the respective NAP (1:200 dilution) for 1 hr at room temperature to label the NAPs. The biofilms were washed twice with PBS and then incubated with goat anti‐rabbit IgG conjugated to AlexaFluor^®^ 594 (Molecular Probes) for 1 hr at room temperature. The biofilms were washed twice in PBS and then incubated with DAPI stain (200 μg/ml) in PBS for 10 min. The biofilms were washed once in PBS and imaged using a x63 objective on a Zeiss 510 Meta‐laser scanning confocal microscope (Carl Zeiss, Thornwood, NY). Three‐dimensional images were reconstructed with AxioVision Rel. 4.8 (Carl Zeiss).

### 2.9 Determination of intracellular steady state level of NAPs in an NTHI biofilm

NTHI strain 86‐028NP biofilm was established on petri dish (100 × 15 mmol/L; Fisher Scientific) for 40 hr. Planktonic cells were removed and the biofilm bacteria were suspended in lysis buffer (20 mmol/L Tris pH 7.4, 1 mmol/L EDTA, 10% glycerol, 20 mmol/L NaCl, 1 mmol/L PMSF, 5 mmol/L MgCl_2_, 5 mmol/L CaCl_2_). The suspension was treated with DNase (100 μg/ml) for 30 min on ice followed by cell lysis by three passes in a French press at 20,000 psi. The cell lysate was clarified at 25,000 g for 30 min at 4°C and passed through a 0.45 μm filter. 50 μg of total soluble protein was resolved in an SDS‐PAGE gel and probed with polyclonal antiserum from rabbits directed against the respective NAP in a western blot analysis. ImageQuant software (GE Healthcare) was employed to quantify the level of NAPs. All in vitro biofilm assays were repeated a minimum of three times on separate days.

### 2.10 Electrophoretic mobility shift assay

HJ DNA was generated from the following oligos: 5′‐Biotin ‐ G*G*AACCTTGGCCTTAACCAACCAAGGTTCCGGTTAAGG*A*A ‐ 3′, 5′ – G*C*AACGTGTGCCGTTAACGAACCTAGGATGGGCATTAGG*T*A – 3′, 5′ – T*T*CCTTAACCGGAACCTTGGTTCGTTAACGGCACACGTT*G*C – 3′ and 5′ – T*A*CCTAATGCCCATCCTAGGTTGGTTAAGGCCAAGGTT*C*C – 3′. Asterisk between two bases indicates that the linkage is a phosphorothioate diester. Equimolar concentration of the oligos were mixed and heated to 95°C for 10 min and then slowly cooled to room temperature to make HJ DNA. The appropriate protein was incubated with Holliday junction (HJ) DNA in reaction buffer (50 mmol/L Tris pH 7.8, 60 mmol/L KCl, 100 μg/ml BSA, 200 μmol/L EDTA) for 30 min at room temperature. The reaction mixtures were then separated, using 6% nondenaturing polyacrylamide gel electrophoresis in 0.5X TBE running buffer at 200 V for 2.5 hr. The resolved DNA was stained with GelRed nucleic acid gel stain (Biotium) and imaged with a Molecular Imager Gel Doc™ XR+ (BIO‐RAD).

### 2.11 Ferroxidase activity

All solutions were filter sterilized, purged with nitrogen, and stored in anaerobic balch‐type glass tubes. Here, 10 mmol/L FeSO_4_ stock solution was prepared in the presence of 1 mmol/L HCl for minimal auto‐oxidation. Protein concentrations were determined by BCA assay (Pierce). 50–200 nmol/L of dodecamer DPS or 100 nmol/L BSA (negative control) was added to 50 mmol/L MOPS‐NaOH, pH 7.0 and incubated at 37°C for 10 min. FeSO_4_ was added to the reaction mixture at a final concentration of 400 μmol/L. The increase in optical density at 305 nm was monitored by a Synergy H1 hybrid plate reader spectrophotometer (BioteK) for 5 min at 37°C.

### 2.12 Statistical evaluation

Statistical significance was assessed by unpaired *t*‐test (GraphPad Prism version 6.0). A *p *≤* *.05 was represented as *, a *p* ≤ .01 was represented by **, and a *p* ≤ .001 was represented by ***.

## 3 RESULTS

### 3.1 NAPs are present within the ECM of biofilms formed in vitro by NTHI

**Figure 1:**
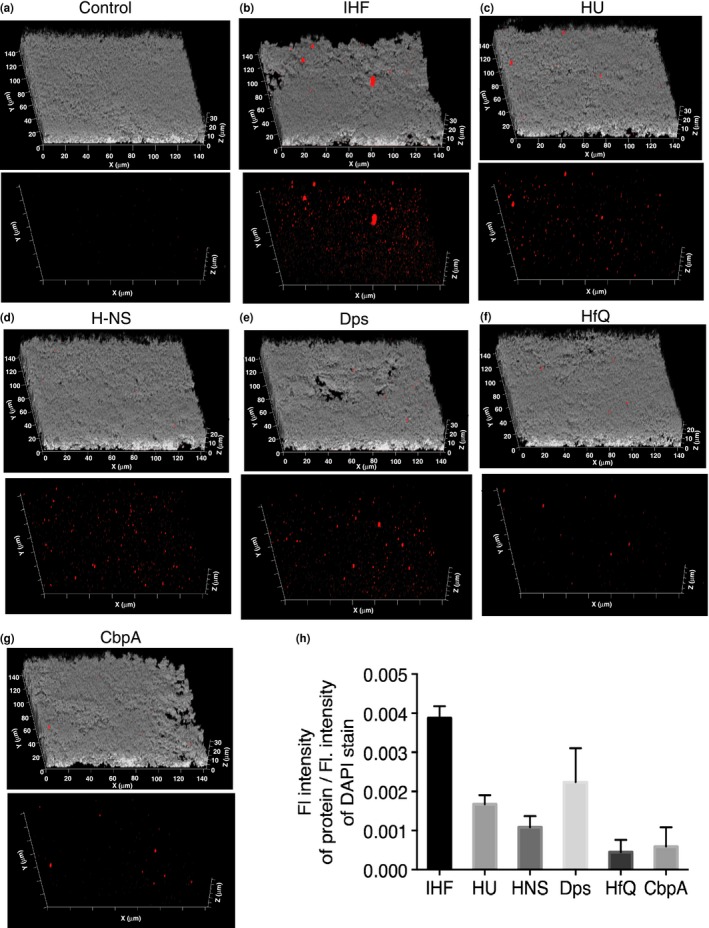
Distribution of NAPs within the ECM of an NTHI biofilm. Biofilm growth was initiated then maintained for 40 hr. Unfixed 40 hr biofilms were incubated with (a) control preimmune IgG (b) anti‐IHF IgG (c) anti‐HU IgG (d) anti‐H‐NS IgG (e) anti‐Dps IgG (f) anti‐HfQ IgG (g) anti‐CbpA IgG and then incubated with goat anti‐rabbit IgG conjugated to AlexaFluor^®^ 594 (NAPs ‐ red). NTHI were stained with DAPI (gray). The biofilms were then imaged using a 63X objective on a Zeiss 510 Meta‐laser scanning confocal microscope. Images represent the side view of biofilms. Bottom panel in each image set shows the distribution of NAPs in the absence of gray. (h) The relative intensity of NAPs was determined by the ratio of protein (red) to DAPI (gray). The biofilm assays were repeated a minimum of three times on separate days. Data are presented as mean values ± standard error of the mean. Note that all tested NAPs including IHF, HU, H‐NS, HfQ, Dps and CbpA are present within the ECM of biofilms formed by NTHI, albeit at different relative concentrations. ECM, extracellular matrix; NAP, nucleoid associated proteins

In order to examine NTHI biofilms for the presence of additional NAPs, we tested the specificity of each of the anti‐NAP antibodies by Western blot analysis. The anti‐NAPs antibodies were highly specific to their respective protein as shown in Figure S1. Next, immunofluorescence was performed on 40 hr biofilms formed by NTHI strain 86‐028NP to determine which NAPs were present within the ECM. We labeled unfixed biofilms with IgG purified from rabbit polyclonal antiserum directed against each of the following isolated and purified recombinant NAPs: IHF, HU, Dps, H‐NS, HfQ, and CbpA. NAPs were revealed with goat anti‐rabbit IgG conjugated to AlexaFluor^®^ 594 (Figure 1). The biofilm biomass was labeled with DAPI (gray). Each of the tested NAPs was present within the biofilm formed by NTHI strain 86‐028NP as indicated by the distribution of red fluorescence throughout the biofilm (Figure 1a–g). IgG purified from preimmune rabbit served as a negative control (Figure 1a). The relative intensity of NAPs was determined by the ratio of the NAP labeling (anti‐NAP) to the DAPI labeling (a measure of total DNA). It was evident from Figure 1h that the DNABII proteins, IHF and HU and Ferritin‐like protein Dps were the most abundant within the biofilms formed by NTHI strain 86‐028NP. The extracellular steady state level of NAPs within the biofilm matrix, with the exception of IHF, corresponded well with the intracellular steady state levels of the respective protein (Figure S2). These data suggested that multiple NAPs, including IHF, HU, H‐NS, HfQ, Dps and CbpA are present within the ECM of biofilms formed by NTHI strain 86‐028NP, albeit at different relative concentrations as detectable within the limits of the assay presented here.

### 3.2 DNABII proteins, IHF and HU are positioned at distinct locations within the ECM of biofilms formed by NTHI strain 86‐028NP

**Figure 2:**
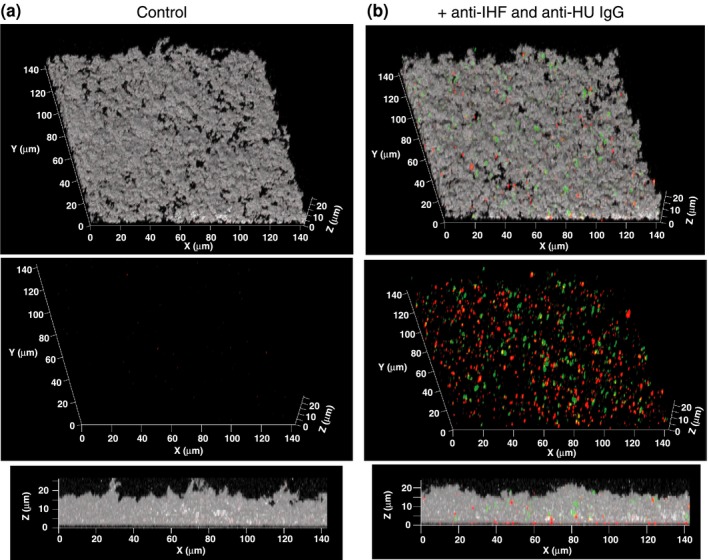
Localization of IHF and HU within an NTHI biofilm. Biofilm growth was initiated and maintained for 40 hr. Unfixed 40 hr biofilms were washed in PBS and incubated with (a) control IgG or (b) anti‐IHFA3, anti‐IHFB2 and anti‐HU_2_ IgG and then incubated with goat anti‐mouse IgG conjugated to AlexaFluor^®^ 405 and goat anti‐guinea pig IgG conjugated to AlexaFluor^®^ 488 as required. NTHI were labeled with FilmTracer^™^ FM ^®^ 4‐64. The biofilms were imaged using a x63 objective on a Zeiss 510 Meta‐laser scanning confocal microscope (Carl Zeiss, Thornwood, NY). NTHI are shown in gray, IHF in red and HU in green. Note that IHF and HU are found at distinct locations within the NTHI biofilm matrix.IHF, integration host factor; PBS, phosphate buffered saline; NTHI, nontypeable *Haemophilus influenzae*

Immunofluorescence was employed to assess the spatial distribution of the two DNABII proteins (IHF and HU) within the biofilm matrix formed by NTHI strain 86‐028NP. Forty‐hour biofilms were incubated with mouse monoclonal antibody directed against the alpha subunit of IHF (IHFA; called ‘anti‐IHFA3′), the beta subunit of IHF (IHFB, called ‘anti‐IHFB2) (Novotny et al., 2016) and polyclonal antibody purified from guinea pig directed against HU peptide. IHF was detected with goat anti‐mouse IgG conjugated to AlexaFluor^®^ 405 (pseudo colored red, Figure 2) and HU was detected with goat anti‐guinea pig conjugated to AlexaFluor^®^ 488 (green, Figure 2). Bacteria resident within the biofilm were labeled with FilmTracer™ FM^®^ 4‐64 (pseudo colored gray, Figure 2). IHF and HU were found at distinct locations within the biofilm matrix as evidenced by the lack of yellow (merge between red and green, Figure 2) within the limits of the assay as presented here. These data suggested either that the two DNABII proteins likely bound to eDNA at different locations or that they have distinct functions within the ECM of biofilms formed by NTHI.

### 3.3 NTHI biofilms are destabilized by treatment with polyclonal antibodies against DNABII proteins, but not upon treatment with antibodies against non‐DNABII proteins

**Figure 3:**
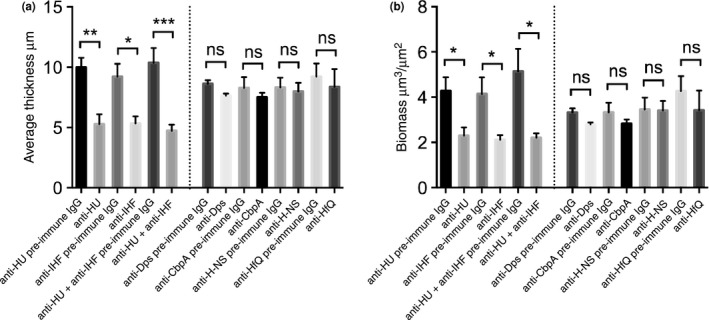
Anti‐IHF and anti‐HU antibodies disrupted preformed NTHI biofilms in vitro. 24‐hr NTHI biofilms were treated with the indicated purified IgG (30 μg) for 16 hr. Biofilms were washed with saline and stained with LIVE/DEAD ^®^ stain. COMSTAT analysis was employed to calculate average thickness (a) and biomass (b). All in vitro biofilm assays were repeated a minimum of three times on separate days. Data are presented as mean values ± standard error of the mean. A *p* ≤ .05 was considered significant. *p *= .01–.05 is represented by *, *p *< 0.01 is represented by **, *p* < .001 is ***. Note significant reduction of biofilm average thickness and biomass is observed only upon treatment with antibodies against DNABII proteins

Since biofilms formed by NTHI strain 86‐028NP incorporate several NAPs (DNABII proteins – IHF and HU; non‐DNABII proteins – Dps, H‐NS‐ HfQ, and CbpA) within the biofilm matrix, we evaluated the role of individual NAPs in maintaining the structural integrity of the biofilms. To this end, we treated 24‐hr biofilms with polyclonal antibodies directed against each NAP individually for 16 hr. Antibody from the respective preimmune rabbit serum was used as a negative control (indicated by preimmune IgG, in Figure 3). Treatment with antibodies against either of the two DNABII proteins, IHF and HU (anti‐HU, anti‐IHF) induced a significant reduction in average thickness (Figure 3a) and biomass (Figure 3b) compared to their respective preimmune antisera (Figure 3a and b). A mixture of antibodies against the two DNABII proteins resulted in no additional reduction in biofilm average thickness or biomass (anti‐HU + anti‐IHF) compared to the addition of each individual anti‐DNABII antibody (Figure 3a and b). It is to be noted that the disruption of biofilms was performed using a single arbitrary concentration of antibody and thereby do not represent the total possible disruptive capability. Strikingly, as evident from Figure 3, incubation with polyclonal antibodies against non‐DNABII proteins (anti‐Dps, anti‐CbpA, anti‐H‐NS, anti‐HfQ) had no effect on biofilm average thickness or biomass compared to their respective preimmune antisera. These results suggested that DNABII proteins were the only major NAPs that were critical to the structural integrity of an NTHI biofilm.

### 3.4 NTHI biofilm structure is intact upon depletion of non‐DNABII NAPs from the biofilm extracellular matrix

**Figure 4:**
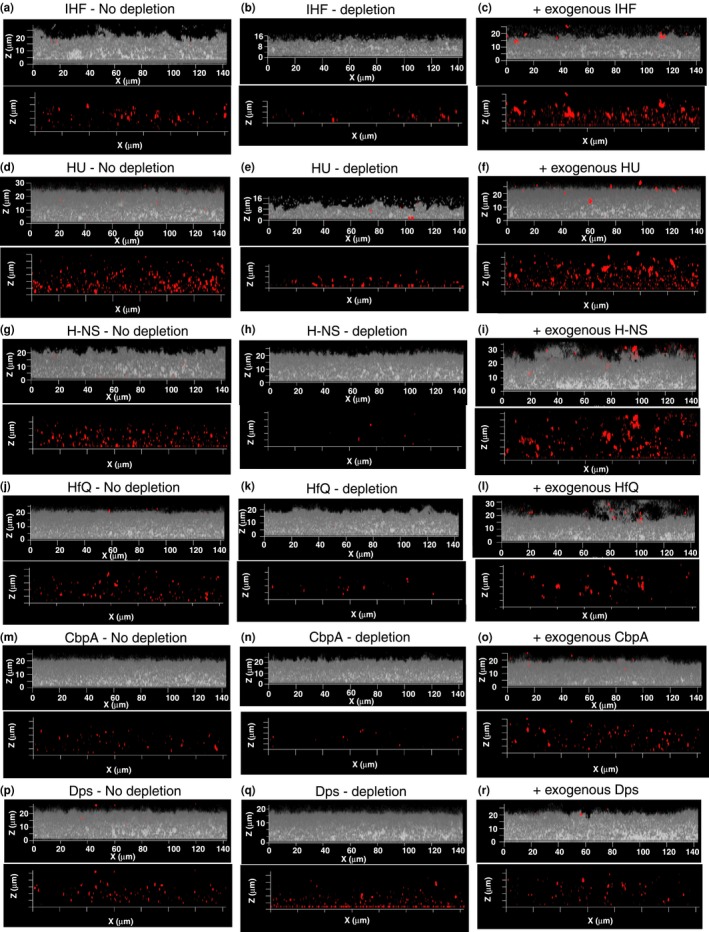
Depletion of NAPs from NTHI biofilm. Biofilm growth was initiated then maintained in the presence of one of the following: (a) respective preimmune serum (No depletion panels – a, d, g, j, m, p) (b) antiserum against the indicated NAP (depletion panels – b, e, h, k, n, q), (c) indicated NAP at 100 nmol/L (panels c, f, i, l, o, r) for 40 hr. Unfixed 40 hr biofilms were washed in PBS and incubated with anti‐IHF antiserum (a, b, c), anti‐HU antiserum (d, e, f), anti H‐NS antiserum (g, h, i), anti‐HfQ antiserum (j, k, l), and anti‐CbpA antiserum (m, n, o), and anti‐Dps antiserum (p, q, r) and then treated with goat anti‐rabbit IgG conjugated to AlexaFluor^®^ 594 (NAPs – red). NTHI biofilms were visualized with DAPI (NTHI – gray). The biofilms were then imaged using a 63X objective on a Zeiss 510 Meta‐laser scanning confocal microscope (Carl Zeiss, Thornwood, NY). Note marked difference in biofilm height only upon depletion of IHF and HU and not upon depletion of non‐DNABII NAPs

The above results demonstrated that of the antibodies that were specific to each NAP, only those directed against IHF or HU disrupted biofilms formed by NTHI strain 86‐028NP. To determine if each of those antibodies truly depleted each of the individual NAPs from the ECM, we performed a combination of antibody treatment followed by immunofluorescence of 40 hr NTHI strain 86‐028NP biofilms. When compared to the biofilms with no depletion (reveals steady state level of NAPs; Figure 4, panels a, d, g, j, m and p), each of the individual NAPs except Dps were depleted from the ECM of NTHI strain 86‐028NP biofilms upon treatment with specific antiserum (Figure 4, panels b, e, h, k, n and q). It should be noted that while the depletion of H‐NS, HfQ and CbpA (non‐DNABII proteins) from the biofilm ECM had no effect on the structural integrity of the biofilm (Figure 4 panels h, k and n), depletion of IHF and HU (DNABII proteins) resulted in a structural collapse of the biofilm (indicated by the reduction in the biofilm height) formed by NTHI strain 86‐028NP (Figure 4b and e) as we have reported previously (Brockson et al., 2014; Goodman et al., 2011; Novotny et al., 2016).

**Figure 5:**
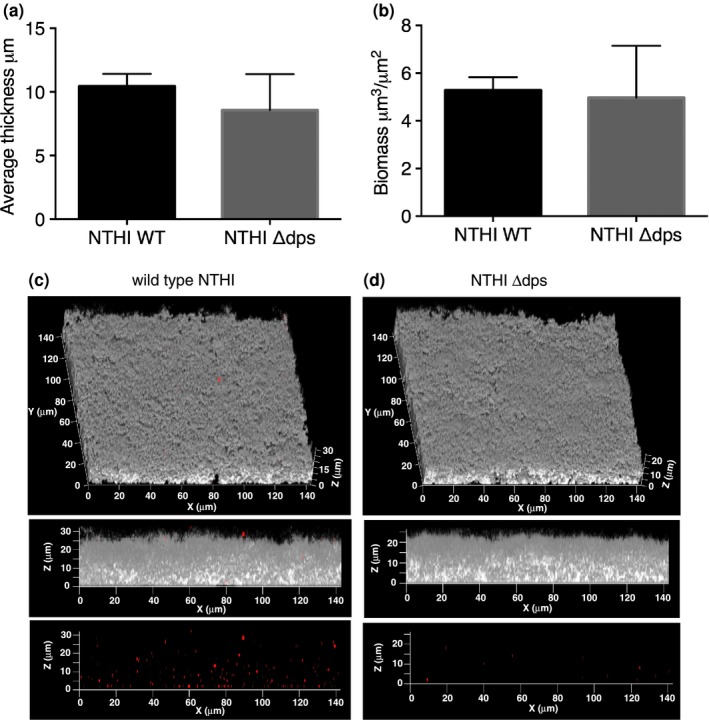
Effect of Dps deficiency on biofilm formation by NTHI. NTHI wild type and NTHI *Δdps* biofilms were grown for 40 hr in vitro in a chamber slide. Biofilms were stained with LIVE/DEAD ^®^ stain and visualized, using a x63 objective on a Zeiss 510 Meta‐laser scanning confocal microscope to acquire 3‐dimensional datasets. Images were analyzed by COMSTAT to calculate average thickness (a) and biomass (b). (c, d) Presence of Dps in the biofilm extracellular millieu of wild type (c) and NTHI Δ*dps* (d) biofilms. Biofilm growth was initiated then maintained for 40 hr. Unfixed 40 hr biofilms were washed in PBS and incubated with polyclonal antiserum from rabbits directed against NTHI Dps and then treated with goat antirabbit IgG conjugated to AlexaFluor^®^ 594. NTHI were labeled with DAPI. The biofilms were imaged using a x63 objective on a Zeiss 510 Meta‐laser scanning confocal microscope (Carl Zeiss, Thornwood, NY). NTHI are shown in gray and Dps is shown in red. Note that the biofilm structure is unaffected in the absence of expression of Dps

Since Dps could not be depleted from the ECM of biofilms formed by NTHI strain 86‐028NP upon treatment with specific antiserum, we employed the wild type NTHI strain 86‐028NP (NTHI WT) and an isogenic strain deficient in Dps (NTHI *Δdps*) in our assay to investigate the role of Dps in NTHI biofilm structure. As shown in Figure 5 panels a and b, the NTHI strain deficient in Dps did not exhibit a defect in biofilm formation compared to WT as evident from similar biofilm average thickness and biomass when grown under the same conditions. The lack of Dps in the ECM of biofilm formed by NTHI strain deficient in Dps was confirmed by immunofluorescence (Figure 5c and d). As a final test, we also exogenously supplemented a 40‐hr biofilm (without predepletion) with 100 nmol/L of each individual recombinant NAP and observed incorporation of the NAPs (indicated by the marked increase in red labeling compared to the ‘no depletion’ panel) within the ECM of biofilms formed by NTHI strain 86‐028NP with no significant changes in biofilm structure (Figure 4, panels c, f, i, l and o). Recombinant Dps when added exogenously could not be incorporated (no marked increase in red labeling compared to panel 4P) within the biofilm ECM of NTHI strain 86‐028NP (Figure 4r), although the recombinant DPS was active as judged by ferrooxidase activity (Figure S3). Since Dps could not be incorporated within the NTHI biofilm ECM, we evaluated the ability of NTHI Dps to bind Holliday Junction DNA (HJ DNA) and found that it was unable to bind to HJ DNA (Figure S4). While the nature of eDNA is not well characterized, HJ DNA is believed to closely represent the interwoven mesh‐like structure of eDNA that has been described for the biofilms formed by several human pathogens (Goodman et al., 2011; Gustave et al., 2013; Idicula et al., 2016; Novotny et al., 2013). *E. coli* Dps binds to DNA via three lysines (K5, K8, and K10) and an arginine residue (R18) in the flexible and disordered N‐terminal tail (Figure S5) (Grant, Filman, Finkel, Kolter, & Hogle, 1998). Members of the Dps family including *Listeria innocua* ferritin, *Bacillus anthracis* Dlp‐1 and Dlp‐2, *Heliocbacter pylori* HP‐NAP, and *Agrobacterium tumefaciens* Dps do not bind to DNA (Ilari, Stefanini, Chiancone, & Tsernoglou, 2000; Papinutto et al., 2002; Yokoyama, Tsuruta, Akao, & Fujii, 2012; Zanotti et al., 2002). The inability of these proteins to bind to DNA was attributed to either the lack of lysines and arginines or shortened positively charged N‐terminal tail. The primary sequence alignment of NTHI Dps to other members of the Dps family revealed that the N‐terminal tail only had a single lysine residue and was shortened by 13 amino acid residues compared to *E. coli* Dps (Figure S5), indicative of the absence of DNA‐binding activity. Collectively, these data suggested that NTHI Dps could not be incorporated within the biofilm matrix likely due to its inability to bind to eDNA. In sum, our results reinforced the concept that IHF and HU are the only major NAPs that play a dominant role in the structural integrity of biofilms formed by NTHI in vitro and also suggested that other NAPs have a negligible role in maintaining the structure of NTHI biofilm.

## 4 DISCUSSION

In this study, we have further demonstrated that the DNABII proteins (IHF and HU) were critical components of the biofilm ECM formed by NTHI and that non‐DNABII NAPs (Dps, H‐NS, HfQ, and CbpA) were dispensable for the structural integrity of an NTHI biofilm in vitro. NTHI encodes eight known NAPs including IHF, HU, Dps, H‐NS, HfQ, Fis, CbpA, and CbpB. CbpB could not be expressed and purified at a quantity sufficient to generate CbpB‐specific polyclonal antiserum and hence was not tested in this study. It has been shown in *E. coli* that Fis is undetectable in stationary phase (Ali Azam, Iwata, Nishimura, Ueda, & Ishihama, 1999), the state during which a biofilm would form and we have further demonstrated that *E. coli* Fis had no effect on Uropathogenic *E. coli* (UPEC) strain UTI89 biofilm structure. Therefore Fis was not tested herein.

NAPs are not known to have a signal peptide for export; however, every tested NAP was detected within the biofilm formed in vitro by NTHI strain 86‐028NP (Figure 1). We have previously demonstrated that DNABII proteins are extracellular, and bind and stabilize the secondary structure of eDNA in the ECM of biofilms formed by Gram‐positive (Rocco et al., 2016) and several Gram‐negative bacteria (Devaraj et al., 2015; Freire et al., 2016; Goodman et al., 2011; Gustave et al., 2013; Idicula et al., 2016; Novotny et al., 2013, 2016), both in vitro and in vivo. The mechanism of extracellular release of NAPs from the bacteria is not well characterized. In this study we have shown that with the exception of IHF, the relative abundance of extracellular NAPs, Dps > HU > H‐NS > CbpA > HfQ (Figure 1h) correspond well with their respective intracellular steady state protein levels, Dps > HU > H‐NS > CbpA > HfQ (Figure S2), suggesting that the release of NAPs into the extracellular milieu is likely stochastic. Recently, it has been demonstrated in NTHI strain 86‐028NP that DNABII proteins are actively exported from the bacterial cell via a Type IV secretion‐like system and ComE pore (Jurcisek, Brockman, Novotny, Goodman, & Bakaletz, 2017). Such an active mechanism of export of DNABII proteins could likely account for the relative abundance of IHF in the extracellular milieu despite its lower expression levels in the intracellular milieu. Cell lysis or autolysis (Claverys & Havarstein, 2007; Rice & Bayles, 2008) could also contribute to the release of NAPs into the extracellular milieu.

We have previously demonstrated that antibodies directed against DNABII proteins disrupt biofilms formed by multiple human pathogens in vitro (Brandstetter et al., 2013; Devaraj et al., 2015; Goodman et al., 2011; Novotny et al., 2013; Rocco et al., 2016), disperse polymicrobial sputum samples recovered from cystic fibrosis patients ex vivo (Gustave et al., 2013), clear NTHI biofilms formed in the chinchilla middle ear during experimental OM, resolve *Pseudomonas aeruginosa* induced lung infection in a murine model, and resolve *Aggregatibacter actinomycetemcomitans* induced peri‐implantitis in vivo (Freire et al., 2011; Novotny et al., 2016). Here we evaluated whether the non‐DNABII NAPs represent potential additional targets for intervention to undermine the structural integrity of NTHI biofilms. While addition of antiserum directed against IHF and HU resulted in depletion of the respective protein from the biofilm matrix and induced a collapse of the biofilm structure, addition of antiserum directed against H‐NS, HfQ or CbpA only resulted in depletion of the respective NAP with no measurable effect on biofilm structure (Figures 3, 4). We have previously demonstrated using a transwell system that treatment of preformed NTHI biofilms (basolateral chamber) with an antibody directed against DNABII proteins (immobilized in the apical chamber), results in collapse of the biofilm despite the fact that antibodies and biofilm never come into contact. We showed that free DNABII proteins were sequestered by the antibodies in the apical chamber and thereby titrating DNABII proteins away from the ECM of the biofilm (Brockson et al., 2014). This sequestration results in a shift in equilibrium of the DNABII proteins from the eDNA‐bound to eDNA‐off state which further destabilizes the biofilm structure. In effect, the antibodies directed against the DNABII proteins are competitive inhibitors of DNABII biding to DNA. Each of the anti‐NAP antibodies were generated against their respective ‘free’ protein and we envision that H‐NS, HfQ and CbpA specific antibodies act in a similar manner by sequestering the respective free/unbound protein, thus causing an equilibrium shift and thereby resulting in depletion of the respective protein from the biofilm ECM, but with no effect on the biofilm structure. These data suggested that although several NAPs are found within the NTHI biofilm ECM, only the DNABII proteins serve as lynchpin proteins to maintain the structure of eDNA whereas other NAPs are dispensable for the structural integrity of the eDNA‐rich ECM. Since the Dps‐specific hyperimmune polyclonal antiserum failed to deplete Dps from the biofilm ECM, we tested an isogenic strain in NTHI deficient in Dps to evaluate its contribution to the biofilm structure. It was intriguing that despite Dps being the most highly expressed NAP in early/late stationary phase (Ali Azam et al., 1999), it had no effect on NTHI biofilm structure (Figure 5); this observation is consistent with Dps' apparent lack of DNA binding function. Collectively, these data suggested that NAPs in the non‐DNABII family do not serve as additional targets for the development of a therapeutic designed to impair the ECM structure of biofilms formed by NTHI.

We have previously shown in UPEC strain UTI89 and *S. gordonii* (Sg) strain Challis that DNABII proteins are limiting and further, that exogenous addition of these proteins resulted in partitioning of the bacteria from the planktonic to the biofilm state which results in increased biofilm size (Devaraj et al., 2015; Rocco et al., 2016). A similar line of investigation was carried out in this study wherein recombinant NTHI NAPs were added exogenously to an NTHI biofilm at seeding. Unlike in UPEC strain UTI89 and Sg, the addition of recombinant NAPs to NTHI strain 86‐028NP had no effect on biofilm structure within the limits of the assay, despite incorporation of the NAPs within the ECM (Figure 4). These data suggested that sufficient quantities of other components of the NTHI ECM are not available to support an increase in the size of the biofilm. NTHI Dps was unable to bind to HJ DNA (Figure S4) and hence was the only NAP that could not be incorporated within the matrix of the biofilm formed by NTHI strain 86‐028NP (Figure 4p and r). NTHI Dps likely serves other functions as in the sequestration of iron and protection of bacteria against oxidative stress within the ECM of an NTHI biofilm.

Most NAPs bind DNA and either bend, wrap or bridge DNA (Dillon & Dorman, 2010). Intracellularly, members of the DNABII family bind to and bend DNA and serve as accessory factors and facilitate replication, transcription, recombination, and repair (Browning et al., 2010). While IHF binds to the consensus sequence WATCAANNNNTTR (where R is a purine and W is an A or T), HU preferentially binds to bent DNA structures like Holliday junctions, three‐way junctions, double‐stranded fork etc., with no apparent sequence specificity (Kamashev & Rouviere‐Yaniv, 2000; Swinger & Rice, 2004). H‐NS is regarded as the genome guardian and universal repressor that binds to curved DNA and facilitates DNA bridges (Azam & Ishihama, 1999). HfQ binds to RNA and curved DNA with no sequence specificity and plays key role in regulating gene expression (Azam & Ishihama, 1999; Vogel & Luisi, 2011). CbpA is a DnaJ homolog that binds to curved DNA in a nonsequence specific manner (Chenoweth & Wickner, 2008). While each of the NAPs bind DNA and play a structural role in the intracellular milieu in shaping the bacterial chromatin, herein we have shown that only the DNABII proteins were critical for maintaining the structural integrity of eDNA rich ECM in vitro. The dominant role of DNABII proteins in maintaining the structure of eDNA is consistent with their universal conservation in Eubacteria and their preference for prebent DNA structural motifs.

In this study, we have demonstrated that IHF and HU were the only NAPs that contributed to the biofilm structure in NTHI in vitro. Although we have previously demonstrated that DNABII proteins stabilize the structure of eDNA by binding at the vertices of crossed strands of eDNA, we had not yet determined the localization of individual DNABII proteins within the eDNA‐rich ECM. Herein, we showed that IHF and HU are not co‐localized within the biofilm (Figure 2), which likely suggests that the two DNABII proteins bind and stabilize eDNA at different locations within the ECM or that these proteins have distinct functions within the NTHI ECM. IHF has two alleles and functions as a dimer. We have previously demonstrated in UPEC strain UTI89 that each subunit of IHF likely forms homodimers and likely have novel functions (Devaraj et al., 2015; Justice et al., 2012). Current investigations include an examination of the spatial distribution and release kinetics of the DNABII proteins with time as well as an analysis of biofilm structures formed by NTHI strains defective in expression of each of the DNABII alleles so as to tease out the specific roles of each of the DNABII proteins in maintaining the structural integrity of eDNA‐rich ECM produced by NTHI.

## CONFLICT OF INTEREST

The authors declare no conflict of interest.
