# Supplementary material for: The DNABII family of proteins is comprised of the only nucleoid associated proteins required for nontypeable Haemophilus influenzae biofilm structure
Source: Microbiologyopen. 2017 Dec 12;7(3):e00563. doi: 10.1002/mbo3.563 (PMC6011942; doi:10.1002/mbo3.563)
Supplement: Supplementary file 1 [file MBO3-7-e00563-s001.pdf]

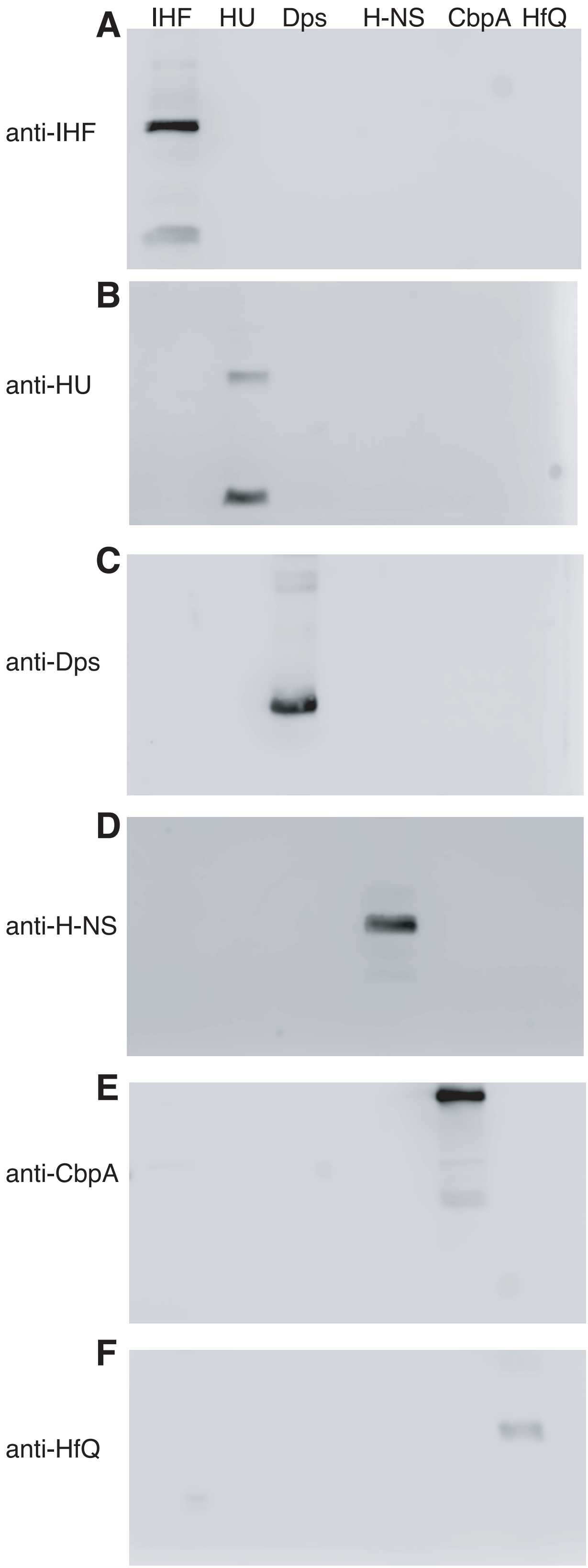

Fig S1. Specificity of anti-NAP antibodies against each of the NAPs.

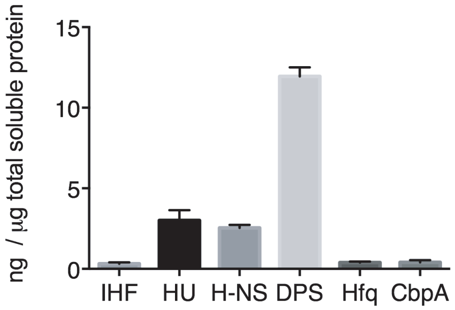

Figure S2. Intracellular steady state level of NAPs in an NTHI biofilm.

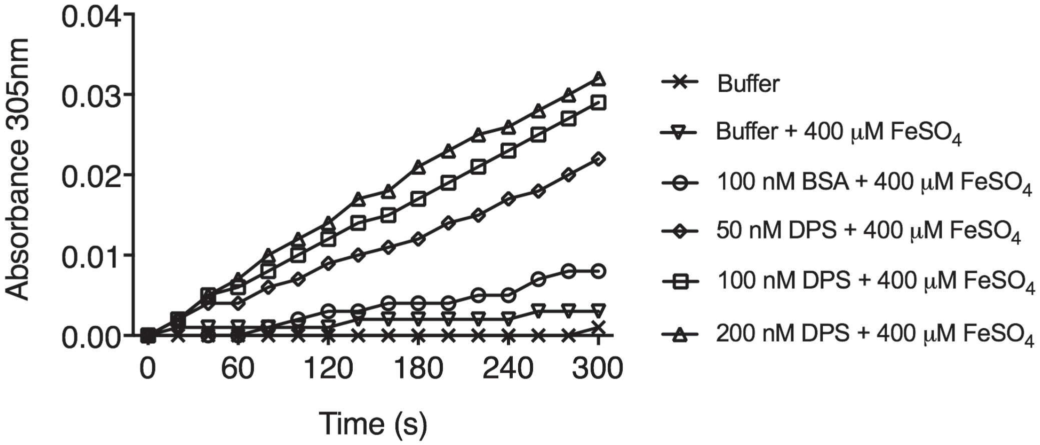

Figure S3. Ferroxidase activity of recombinant NTHI Dps.

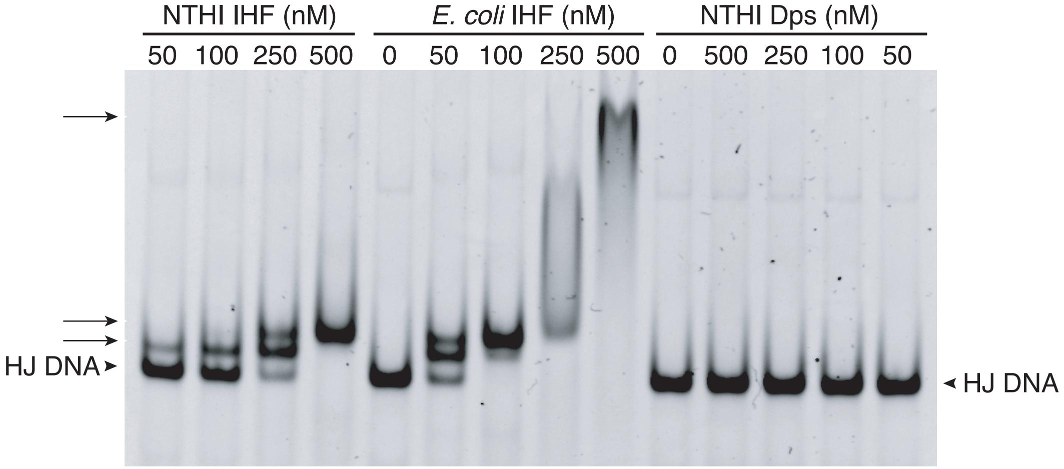

Figure S4. EMSA of NTHI Dps, NTHI IHF and *E. coli* IHF with Holliday junction (HJ) DNA substrate.

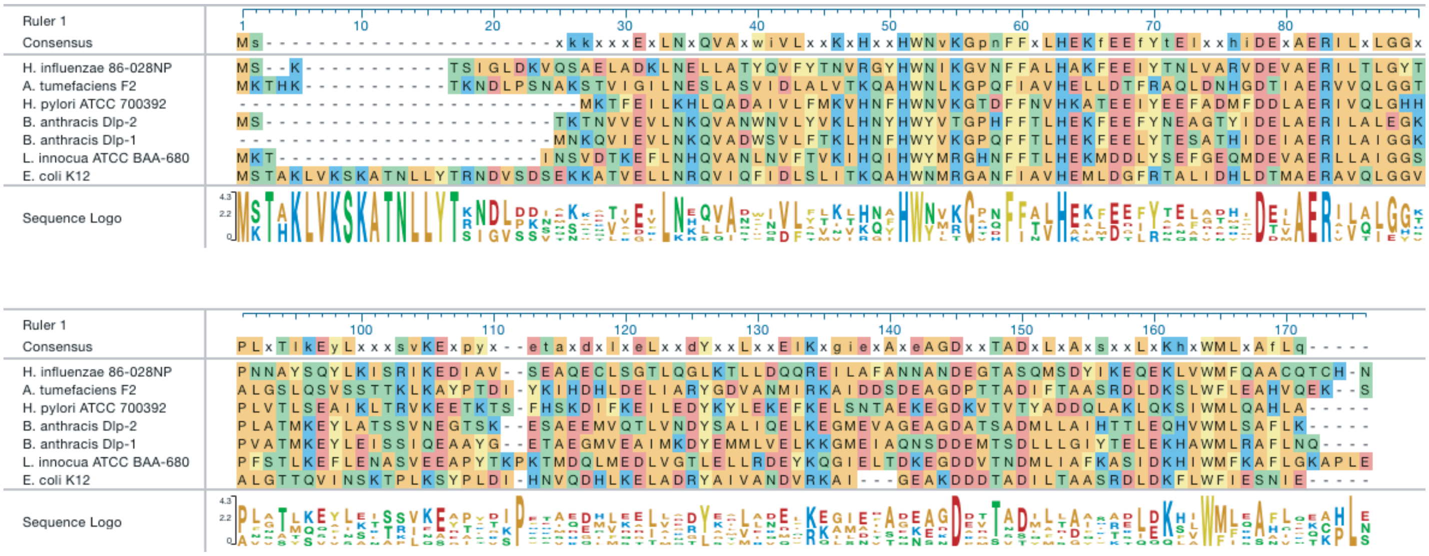

Figure S5. Primary structure alignment of Dps proteins.
